# Quality of life predicts rehabilitation prognosis in Parkinson's disease patients: Factors influence rehabilitation prognosis

**DOI:** 10.1002/brb3.2579

**Published:** 2022-04-16

**Authors:** Detao Meng, Zhaohui Jin, Keke Chen, Xin Yu, Yixuan Wang, Wenjun Du, Jingran Wei, Jianing Xi, Boyan Fang

**Affiliations:** ^1^ Parkinson Medical Center Beijing Rehabilitation Hospital Capital Medical University Beijing China; ^2^ Beijing Rehabilitation Medical College Capital Medical University Beijing China

**Keywords:** health‐related quality of life, Parkinson's disease, PDQ‐39, predictor, rehabilitation

## Abstract

**Background:**

Rehabilitation has been reported to improve the quality of life (QoL) of patients with Parkinson's disease (PD). Nevertheless, not all patients are satisfied with rehabilitation outcomes and could achieve a significant improvement in QoL.

**Objective:**

To detect possible predictors of QoL improvement in patients with PD after rehabilitation.

**Methods:**

A total of 86 PD patients were included and followed up for 3 months with a 39‐item Parkinson's Disease Questionnaire summary index (PDQ‐39 SI) as the primary endpoint. All patients received 2 weeks of multidisciplinary intensive rehabilitation treatment (MIRT). Changes in patients' QoL were assessed using the PDQ‐39 at baseline and at the 3‐month follow‐up. The reliable change index (RCI) was adapted to determine the individual QoL outcome. The predictors of QoL outcome were detected using logistic regression analysis.

**Results:**

After a 3‐month follow‐up, PDQ‐39 SI decreased significantly from 22.95 ± 9.75 to 18.73 ± 10.32 (*P* < 0.001). Scores for QoL improved (RCI>10.9) after rehabilitation for 18.6% of the patients, and 74.4% of patients reported an unchanged QoL (−10.9≤RCI≤10.9), while 7.0% of patients reported a worsening of QoL (RCI<−10.9). Among the baseline parameters, the PDQ‐39 SI was a baseline predictor for changes in QoL in the logistic regression model (OR: 1.15, CI: 1.07–1.24, *P* < 0.001).

**Conclusions:**

MIRT could improve QoL for some patients with PD, and PDQ‐39 score at baseline is the most important predictor for QoL improvements after rehabilitation for this patients.

## Graphical abstract

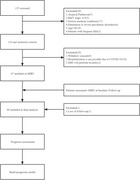

## 1 INTRODUCTION

Parkinson's disease (PD) is a progressive neurodegenerative disease that is characterized by motor symptoms such as bradykinesia, resting tremor, rigidity, and a sequence of nonmotor symptoms, such as cognitive decline, anxiety, and depression (Bloem et al., 2021). It can lead to a decline in quality of life (QoL) very early and deteriorates as the disease progresses (Carod‐Artal et al., 2007), even under the best medical treatment or with deep brain stimulation (Daniels et al., 2011). Therefore, rehabilitation therapies are considered an adjuvant to pharmacological and surgical treatments to maximize functional abilities and to minimize secondary complications (Armstrong & Okun, 2020). Meanwhile, rehabilitation has also been reported to improve the QoL related to PD (Clarke et al., 2016; Ferrazzoli et al., 2018; Monticone et al., 2015; Morris et al., 2009; Rodrigues De Paula et al., 2006; Tickle‐Degnen et al., 2010), but not all studies yield the same results (Wade et al., 2003). Although several reasons could explain the lack of benefit, such as previously satisfactory management leaving little room for improvement, lack of psychological intervention, etc. (Playford, 2003). Therefore, this revealed that not all patients are satisfied with the rehabilitation results and can achieve a significant improvement in QoL.

The reasons may be multidimensional, but strategies to identify which patient could benefit from rehabilitation seem to be important for optimized treatment outcomes. For instance, we can adopt different rehabilitation strategies for patients with different outcomes to reduce the waste of medical resources and improve the therapeutic effect of individual patients. On the other hand, for those who may benefit less, other treatment methods should be actively adopted to improve the patient's QoL as much as possible.

Therefore, we performed this study to understand the effects of multidisciplinary intensive rehabilitation treatment (MIRT) for mild to moderate PD patients. In particular, the relative contributions of motor and nonmotor baseline parameters to the rehabilitation outcome on QoL were analyzed. We attempted to detect the possible predictors of QoL improvement for PD patients after rehabilitation with a 3‐month follow‐up.

## 2 METHODS

### 2.1 Study population

In this ongoing, prospective single‐center cohort study (multidisciplinary rehabilitation registration study on PD, registration number: ChiCTR2000033768), a total of 86 PD patients attending our inpatient rehabilitation project in Beijing Rehabilitation Hospital from June 2020 to July 2021 were included and followed up for 3 months. All patients received 2 weeks of MIRT. None of the patients underwent drug adjustment from training to 3 months of follow‐up. Inclusion criteria were: (1) idiopathic PD as confirmed by a neurologist using the Movement Disorder Society criteria (Postuma et al., 2015); (2) no deep brain stimulation or in vivo implantation treatment; (3) were able to understand each item of the informed consent and willing to sign the informed consent. The exclusion criteria were as follows: (1) atypical parkinsonism, such as multiple system atrophy, corticobasal degeneration, and progressive supranuclear palsy; (2) serious medical conditions, such as severe coronary heart disease, backache, and malignancy; and (3) individuals with dementia or severe psychiatric symptoms. (4) Hoehn & Yahr (H&Y) Stage: 4–5; (5) patients with frequent falls; (6) age > 80.

This research was approved by the ethics committee of the Beijing Rehabilitation Hospital (2020bkky010). All participants signed informed consent following the Declaration of Helsinki. Clinical and demographic data at baseline collected included sex, age, age at diagnosis, disease duration, levodopa equivalent daily dose (LEDD), H&Y stage (Hoehn & Yahr, 1967), and MDS‐UPDRS score (Goetz et al., 2007). The evaluation of the H&Y stage and MDS‐UPDRS score were carried out under the medication ON stage.

#### 2.1.1 MIRT procedure

MIRT is specifically designed for rehabilitation for PD patients and is constituted with a multidisciplinary, aerobic, motor‐cognitive, intensive, and goal‐based rehabilitation treatment (Ferrazzoli et al., 2018; Frazzitta et al., 2012; Frazzitta et al., 2015). All patients received a 2 weeks of MIRT. The 2‐week program comprised four sessions. The first session is one‐on‐one physical therapy by a physical therapist for 30 min. The second session is a 60‐min goal‐directed balance and gait training combined with motor‐cognitive dual tasks by an augmented reality treadmill (C‐MiLL, Motek, Amsterdam/Culemborg, Netherlands) and Balance Tutor (Meditouch, Netanya, Israel). The third session is a 30‐min aerobic training on an upper and lower limb trainer (T5XR; Nustep, Ann Arbor, MI, USA) and the fourth session is 30‐min speech therapy. All the training was conducted in a hospital setting, 5 days per week, and the details were described elsewhere (Chen et al., 2021).

### 2.2 Clinical assessments

QoL was assessed by the Chinese version of the Parkinson's Disease Questionnaire (PDQ‐39) (Neff et al., 2018). The PDQ‐39 is a validated disease‐specific HRQoL measure in PD (Neff et al., 2018) that contains eight domains: mobility, activities of daily life, emotional well‐being, stigma, social support, cognition, communication, and bodily discomfort. The PDQ‐39 summary index (PDQ‐39 SI) was calculated to measure overall health‐related QoL, and a higher score (range 0–100) indicates a more severe burden of QoL. The outcome parameters were the PDQ‐39 SI. The differences between the follow‐up 3 months after rehabilitation and baseline scores were calculated as outcome parameters. The Reliable Change Index (RCI) was adapted to determine the individual outcome of QoL after rehabilitation (Daniels et al., 2011; Jacobson & Truax, 1991). A PDQ‐39 score decrease of more than 10.9 points was termed “improved,” and a score increase of more than 10.9 points was termed “worsen”; changes between −10.9 and 10.9 were termed “unchanged” (Daniels et al., 2011; Floden et al., 2014; Liu et al., 2019; Witt et al., 2011). Both the “worsen” and “unchanged” groups were assigned to the “nonimproved” group.

Furthermore, cognitive function was evaluated by the Montreal Cognitive Assessment (MoCA) (Nasreddine et al., 2005), depression was evaluated by the Geriatric Depression Scale (GDS) (Montorio & Izal, 1996), fatigue was evaluated by the Parkinson Fatigue Scale (PFS⁃16) (Brown et al., 2005), and apathy was evaluated by the Modified Apathy Evaluation Scale (MAES) (Starkstein et al., 1992) at baseline as a factor that affects QoL according to the literature (D'Iorio et al., 2017; Rahman et al., 2008; Rahman et al., 2008; Tang et al., 2020).

### 2.3 Statistical analysis

Data distribution and normality were evaluated with the Shapiro–Wilk test. Normally distributed data are expressed as the mean ± standard deviation (SD), while nonnormally distributed data are reported as the median (interquartile range). The PDQ‐39 SI and subgroup score at baseline and with follow‐up for 3 months were compared by the paired sample *t* test. Spearman correlations or Pearson correlations were used to explore the relationship between changes in QoL scores and demographic and clinical parameters, including age, age at diagnosis, disease duration, LEDD, H&Y stage, MDS‐UPDRS score, GDS, MAES, PFS‐16, and PDQ‐39 SI score at baseline. Spearman's correlation was also used to evaluate changed nonmotor parameters, including GDS, MAES, and PFS‐16 scores, associated with changes in QoL.

Furthermore, demographic and clinical parameters at baseline were compared between the “improved” and “nonimproved” groups according to the PDQ‐39 RCI using univariate analysis. Demographic and clinical parameters at baseline as revealed by the first‐step analyses (less strictly selected: *P* < 0.10, one‐tailed) were included in a logistic regression analysis as independent variables with the dichotomous variable improved/nonimproved in terms of the PDQ‐39 RCI as the dependent variable. *P* values < 0.05 (two‐tailed) were regarded as significant. Receiver operating characteristic (ROC) analyses were used to evaluate the predictive utility of logistic regression models on patients’ QoL improvement (Schrag et al., 2017). These statistical data were analyzed using SPSS version 21 (SPSS Inc., Chicago, IL).

## 3 RESULTS

**FIGURE 1:**
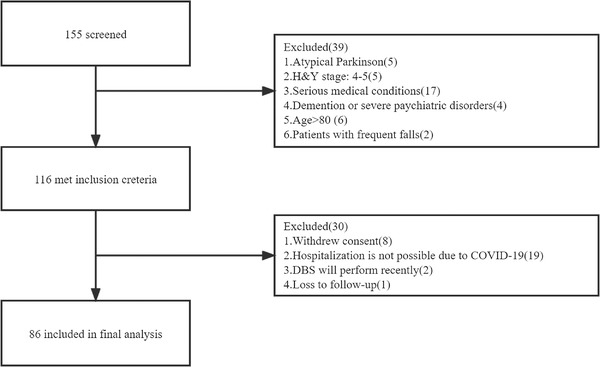
Participants flow chart. The flow chart describes the enrollment of patients

Of 155 patients screened, 86 patients (36 males) were included in the final analysis (see Figure 1). The mean age at baseline was 60.24 years (SD = 7.82), and the median disease duration was 6.00 years (interquartile range = 4.00). The patient baseline characteristics are shown in Table 1.

**TABLE 1:** Patient characteristics at baseline

	Baseline
Male^a^, n (%)	36 (41.86%)
Age at onset^b^ (y)	54.00 (9.00)
Age^c^ (y)	60.24 ± 7.82
Disease duration^b^ (y)	6.00 (4.00)
LEDD^b^ (mg/d)	520.50 (362.50)
Jankovic subtype^a^, n (%)	
Tremor dominant	31 (36.1%)
Postural instability/gait dominant	48 (55.8%)
Indeterminate	7 (8.1%)
Hoehn and Yahr Stage^b^	2.00 (0.50)
UPDRS part I score^b^	8.00 (5.50)
UPDRS part II score^c^	11.81 ± 6.21
UPDRS part III score^c^	32.62 ± 13.73
UPRDS part IV score^b^	2.00 (5.00)
MoCA^c^	25.20 ± 4.37
GDS^b^	8.00 (10.00)
MAES^b^	11.00 (15.00)
PFS‐16^c^	42.45 ± 13.88
PDQ‐39 SI^c^	22.95 ± 9.75

GDS, Geriatric Depression Scale; LEDD, levodopa equivalent daily dose; MAES, Modified Apathy Evaluation Scale; MoCA, Montreal Cognitive Assessment; PDQ‐39 SI, Parkinson's Disease Questionnaire 39‐summary index; PFS⁃16, Parkinson Fatigue Scale; UPDRS, Unified Parkinson's Disease Rating Scale.

^a^ Count data are expressed as n (%).

^b^ Non‐normal data are reported as the median (interquartile range).

^c^ Normally distributed data are expressed as the mean ± SD.

### 3.1 The effects of MIRT for mild to moderate PD patients

**FIGURE 2:**
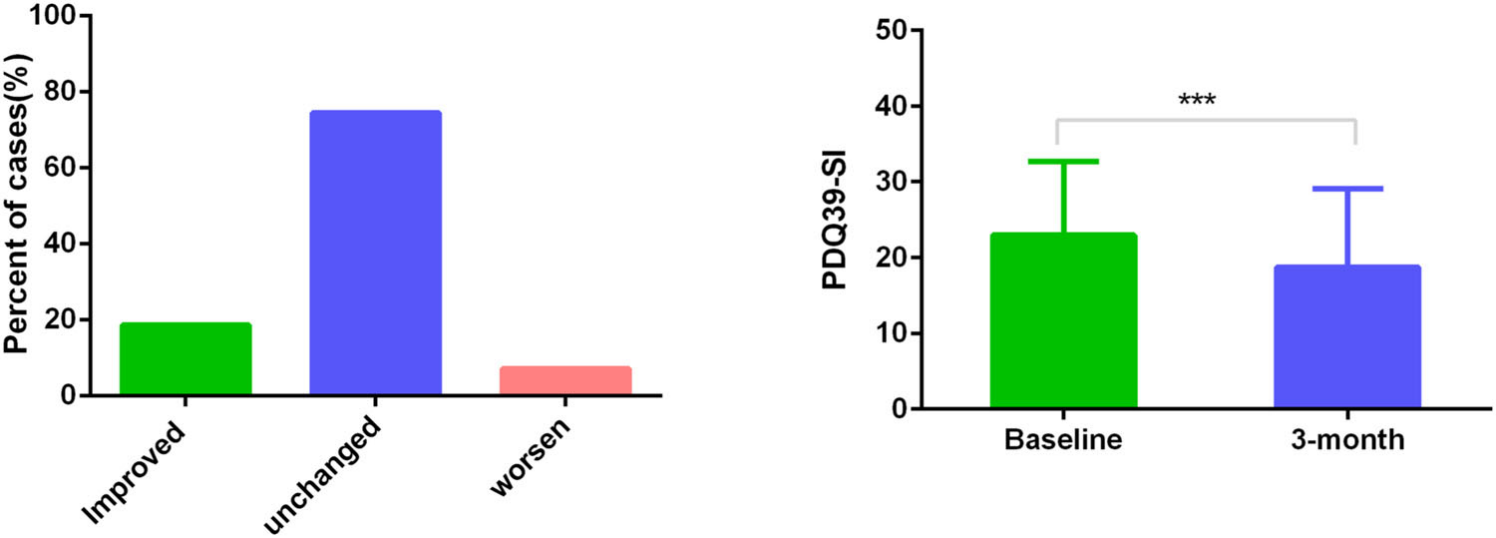
The effects of MIRT for mild to moderate PD patients. Left: For the PDQ‐39 SI, improved, unchanged, and worsened groups are shown in the percent of cases. QoL improved in 18.6% of patients, 74.4% of patients had no changes in QoL, QoL deteriorated in 7.0% of patients. Right: The PDQ‐39 SI at baseline decreased significantly from baseline to the 3‐month follow‐up. (PDQ‐39 SI: PD Questionnaire‐39 summary score, *P < 0.05). MIRT, multi‐disciplinary intensive rehabilitation treatment

After a 3‐month follow‐up, scores for QoL improved (RCI > 10.9) after rehabilitation for 18.6% of the patients (16 of 86 patients) and were assigned to the “improved” group. 74.4% of patients (64 of 86 patients) reported an unchanged QoL(−10.9≤RCI≤10.9), and 7.0% of patients (6 of 86 patients) reported a worsening of QoL(RCI<−10.9). Both of them were assigned to the “nonimproved” group. The PDQ‐39 SI at baseline was 22.95 ± 9.75, and it decreased significantly to 18.73 ± 10.32 (*P* < 0.001) with a 3‐month follow‐up. The overall effects of MIRT for mild to moderate PD patients are shown in Figure 2.

**FIGURE 3:**
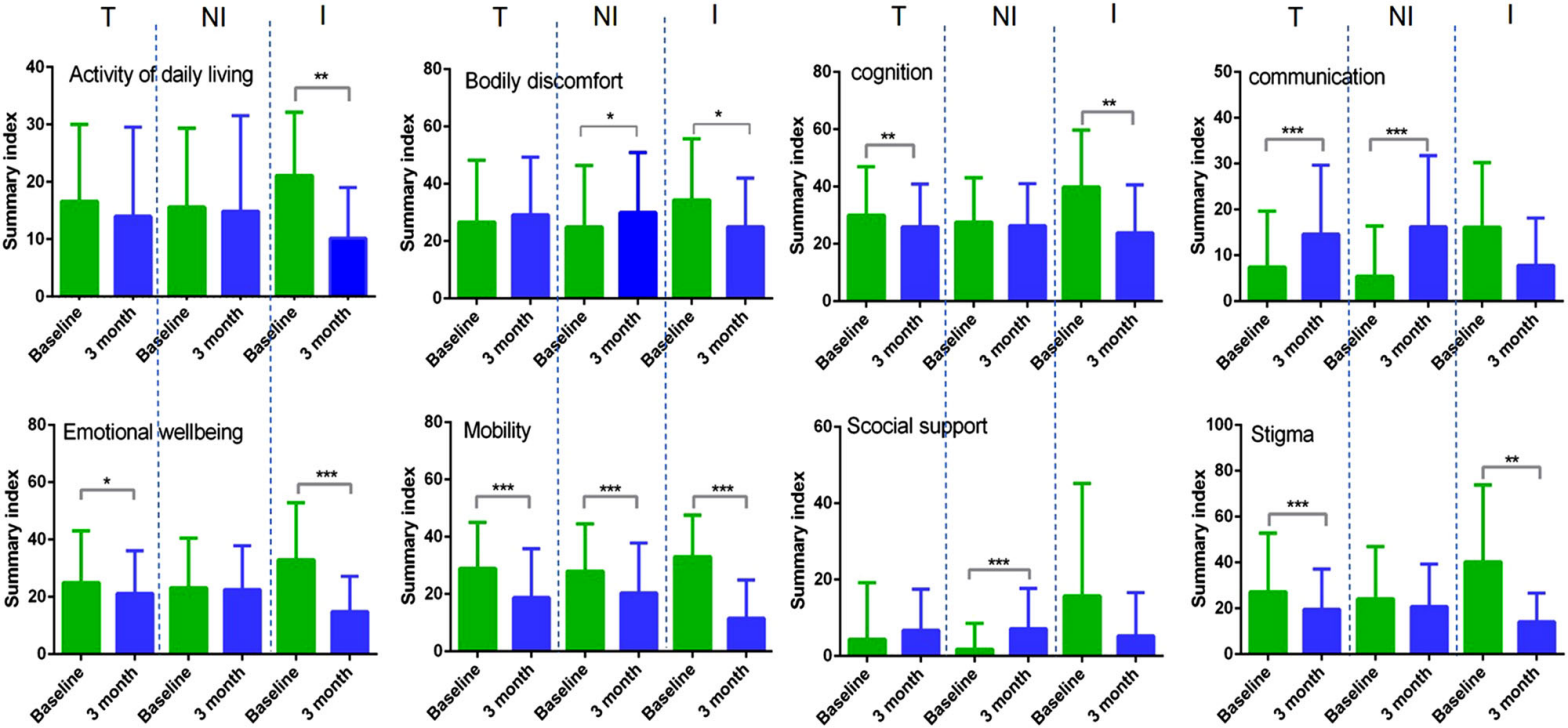
The effects of MIRT for mild to moderate PD patients in the PDQ‐39 subgroup. The comparison of PDQ‐39 subgroup scores for all patients, the unimproved group, and the improved group at baseline and after rehabilitation is separately shown on the left of the dotted line, between the two dotted lines, and on the right of the dotted line. The cognition, stigma, emotional wellbeing, and mobility domains decreased significantly, and the communication domain increased significantly. In the “improved” group, bodily discomfort, cognition, stigma, emotional wellbeing, activities of daily living, and mobility domains decreased significantly. In the “nonimproved” group, the mobility domain decreased significantly, and there was no change or increase significantly in other domains (T, total; NI, nonimproved; I, improved. *P < 0.05, **P < 0.01, ***P < 0.001). MIRT, multi‐disciplinary intensive rehabilitation treatment

In the PDQ‐39 subgroup, the cognition (from 29.87 ± 16.95 to 25.87 ± 15.01, *P* = 0.008), stigma (from 27.03 ± 25.77 to 19.55 ± 17.59, *P* < 0.001), emotional wellbeing (from 24.90 ± 18.13 to 21.07 ± 14.98, *P* = 0.035), and mobility (from 28.87 ± 16.17 to 18.69 ± 17.07, *P* < 0.001) domains decreased significantly; however, the communication domain increased significantly from 7.36 ± 12.29 to 14.63 ± 15.01 (*P* < 0.001). Bodily discomfort, social support, and activities of daily living showed no significant changes. In the “improved” group, the bodily discomfort (*P* = 0.029), cognition (*P* = 0.002), stigma (*P* = 0.002), emotional wellbeing (*P* < 0.001), activity of daily living (*P* = 0.002), and mobility (*P* < 0.001) domains decreased significantly. There is a downward trend in the social support and communication domain. In the “nonimproved” group, the mobility domain decreased significantly (*P* < 0.001); however, there was no significant change or increase in other domains. The PDQ‐39 subgroup analysis was shown in Figure 3.

### 3.2 Correlation analyses

PDQ‐39 SI at baseline was significantly associated with changes in the PDQ‐39 SI after a 3‐month follow‐up (*r* = 0.422, *P*<0.001). Meanwhile, the changes in the PDQ‐39 SI were significantly associated with GDS score changes (*r* = 0.537, *P* < 0.001) and MAES score changes (*r* = 0.284, *P* < 0.01) after a 3‐month follow‐up. The correlation analysis results are shown in Appendix 1.

### 3.3 Logistic regression analysis

**FIGURE 4:**
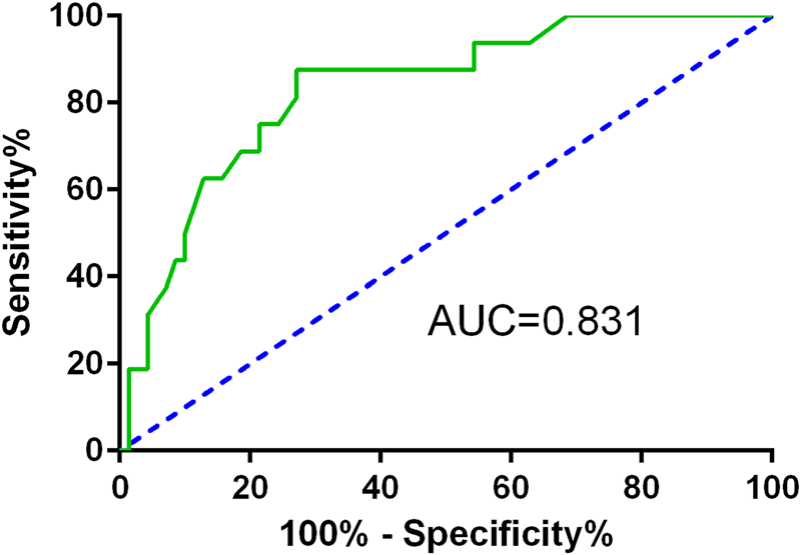
Receiver operating characteristic (ROC) curve for the model (AUC = 0.831, *P*<0.001). ROC curve demonstrating the classification accuracy (predicted probability of “improved” vs “nonimproved”) of the logistic regression. The diagonal dashed line represents chance classification accuracy

Table 2 includes unadjusted ORs for bivariable analyses and adjusted ORs for multivariate analyses assessing the magnitude of association between each candidate predictor and the outcome. GDS (*P* = 0.013) and PDQ‐39 SI (*P*<0.001) at baseline showed significant relationships with the outcome. For every additional GDS score, patients displayed a 12.5% higher odds of improvement in QoL (OR = 1.125, 95% confidence interval [CI] 1.025‐1.235, *P* = 0.013). For every additional score of the PDQ‐39, patients displayed a 15.1% higher odds of improvement in QoL (OR = 1.151, 95% CI 1.067‐1.241, *P* < 0.001). The multivariate regression model included three factors at baseline with *P* < 0.1 in the univariate analysis: PDQ‐39 SI (*P* < 0.001), GDS (*P* = 0.013), and age (*P* = 0.098). Only the PDQ‐39 SI remained significant (*P* < 0.001) as a baseline predictor for change in QOL in the multivariate model with the likelihood ratio method. The PDQ‐39 SI at baseline was included in the final model. In the final model, for every additional score of the PDQ‐39, patients displayed a 15.1% higher odds of improvement in QoL (OR = 1.151, 95% CI 1.067‐1.241, *P* < 0.001). The area under the ROC curve (AUC) for this model was 0.83 [0.726‐0.935, *P* < 0.001]. The ROC curve for the model is displayed in Figure 4.

**TABLE 2:** Univariate and multivariate analysis of predictors of PDQ‐39 after 3 months

Variables	Univariate analysis		Multivariate analysis	
	OR (95% CI)	*P* value	OR (95% CI)	*P* value
Male/female	2.048(0.682–6.144)	0.201		
Age at onset (y)	1.037(0.965–1.114)	0.320		
Age (y)	1.070(0.987–1.160)	0.098		
Disease duration (y)	1.058(0.948–1.180)	0.317		
LEDD(mg/d)	1.000(0.999–1.002)	0.619		
Jankovic subtype, n (%)				
Tremor dominant	1			
Postural instability/gait dominant	1.750(0.179–17.080)	0.630		
Indeterminate	1.200(0.127–11.374)	0.874		
Hoehn and Yahr Stage	0.829(0.265–2.592)	0.747		
UPDRS part I score	1.052(0.950–1.165)	0.327		
UPDRS part II score	1.004(0.919–1.096)	0.929		
UPDRS part III score	0.980(0.941–1.022)	0.344		
UPRDS part IV score	0.941(0.771–1.148)	0.547		
MoCA	1.043(0.899–1.211)	0.576		
MAES	1.046(0.980–1.117)	0.176		
PFS‐16	1.013(0.974–1.054)	0.511		
GDS	1.125(1.025–1.235)	0.013*		
PDQ‐39	1.151(1.067–1.241)	<0.001*	1.151(1.067–1.241)	<0.001*

GDS, geriatric depression scale; LEDD, levodopa equivalent daily dose; MAES, Modified Apathy Evaluation Scale; MoCA, Montreal Cognitive Assessment; PDQ‐39: Parkinson's Disease Questionnaire‐39; UPDRS, Unified Parkinson's Disease Rating Scale; PFS⁃16, Parkinson Fatigue Scale.

* *P* < 0.05.

## 4 DISCUSSION

Consistent with previous reports (Ferrazzoli et al., 2018; Monticone et al., 2015; Rafferty et al., 2017), our findings suggest that in mild to moderate PD, MIRT could improve QoL for some patients with PD. Although several studies have suggested that rehabilitation treatment, such as MIRT, could effectively improve QoL in patients with PD (Ferrazzoli et al., 2018; Monticone et al., 2015; Oguh et al., 2014), our study showed that the same is not true for all PD patients. QoL improved steadily in only a fraction of patients with a relatively conservative index to define the change in QoL after rehabilitation (Daniels et al., 2011). Therefore, we developed a model that could be used to predict improvement in QoL after rehabilitation in PD patients. We found that impaired QoL was the most important predictor of benefit in mild to moderate PD after rehabilitation in our prognostic model. Furthermore, we found that changes in the PDQ‐39 SI were significantly associated with PDQ‐39 SI at baseline and were significantly associated with GDS score changes and MAES score changes.

Evidence of the positive effects of rehabilitation treatments in PD is increasing (Clarke et al., 2016; Ferrazzoli et al., 2018; Monticone et al., 2015; Morris et al., 2009; Rodrigues De Paula et al., 2006). Ferrazzoli et al.’s study (Ferrazzoli et al., 2018) suggested that MIRT, which consisted of a multidisciplinary, aerobic, intensive, and motor‐cognitive rehabilitation treatment, could improve QoL in patients with PD that could last for 3 months (Ferrazzoli et al., 2018). Monticone et al.’s study (Monticone et al., 2015) found that PD patients could obtain a benefit from multidisciplinary rehabilitative care for at least 1 year after the intervention. Multidisciplinary rehabilitative care could change the course of motor impairment, balance, activities of daily living, and QoL. Morris et al.’s study (Morris et al., 2009) suggested that inpatient rehabilitation produces short‐term reductions in disability and improvements in QoL in people with PD for at least 3 months. As mentioned above (Ferrazzoli et al., 2018; Monticone et al., 2015; Morris et al., 2009), our finding also suggest that the improvement of QoL was considered to be significant by MIRT in mild to moderate PD patients. QoL scores significantly improved for 18.6% of the patients with a more conservative method (Daniels et al., 2011). Another study showed that QoL improved in only 7% of patients who received the best medical treatment with the same definition of improvement (Daniels et al., 2011). In our PDQ‐39 subgroup analysis, we also found that regardless of whether QoL improves, there are significant improvements in the mobility domain. The patients in the “improved” group obtained varying degrees of improvement in all PDQ‐39 subgroup scores. Therefore, our study demonstrated that rehabilitation therapy is an effective adjuvant to pharmacological and surgical treatments to improve QoL in PD patients (Abbruzzese et al., 2016).

Schuepbach et al.’s study showed that impaired QOL is the most important predictor of benefit in patients with PD after deep brain stimulation of the subthalamic nucleus (Schuepbach et al., 2019). Although the principles and mechanisms of DBS are different from those of rehabilitation (Abbruzzese et al., 2016; Ashkan et al., 2017), such advanced research is worthy of our study and emulation. In our study, we assembled a model that predict the improvement of QoL who attended our inpatient rehabilitation. We found that the PDQ‐39‐SI at baseline was a significant predictor of QoL improvement and a heavier burden of QoL at baseline correlated with a better QoL improvement, which is consistent with Ritter's study (Ritter & Bonsaksen, 2019). Ritter's study also found that patients who have lower levels of initial QoL benefit more from rehabilitation. Furthermore, our model's accuracy was confirmed by ROC curves with an AUC = 0.83. Although these results need confirmation in further studies, the results still have a certain guiding value for the choice of therapeutic strategies for PD patients.

Why is impaired QoL the most important predictor of benefit in patients with PD after rehabilitation? In our research, a worse QoL at baseline predicted improvement of QoL in mild to moderate PD patients. One possible reason is some patients had already had a good QoL, lead to limited room left for the improvement. Although patients' QoL has been improved, but they cannot reach the RCI index criterion. Another possible reason is that patients with poor QoL tend to have more severe motor and nonmotor symptoms, which are the determinants of QoL in patients with PD (Martinez‐Martin, 2017; Rahman et al., 2008). Rehabilitation therapy could improve the motor and nonmotor symptoms of PD (Lamotte et al., 2015; Rafferty et al., 2017), and consistent with previous reports (Rafferty et al., 2017), patients with advanced PD could obtain more benefit than those with mild PD from rehabilitation therapy.

There are some limitations to this study that must be acknowledged. First, an important limitation of our findings is the selected patient population. We collected only mild to moderate PD patients, which might lead to selection bias. PD patients in the H&Y:4‐5 stage could not complete MIRT due to their serious condition, and they may need to adopt other rehabilitation strategies, so we did not include those patients. Second, due to the COVID‐19 outbreak, patients were unable to get to the hospital for a detailed kinematic evaluation, such as UPDRS‐III score and UPRDS part IV score, at the 3‐month follow‐up, which could reflect the improvement of the patient's motor function and motor complications (Goetz et al., 2007). Last, our follow‐up time was only 3 months. Although there is evidence that PD patients can benefit from rehabilitation treatments, it is unclear how long the benefits could last (Abbruzzese et al., 2016). A long‐term observation still is needed.

In conclusion, the present study shows that MIRT could improve the QoL of PD patients, especially mild to moderate PD patients with impaired QoL. Impaired QoL at baseline could predict the benefit of rehabilitation for PD patients. Patients with impaired QoL should actively participate in rehabilitation exercises to improve their QoL. Further research is needed in more PD patients.

## CONFLICT OF INTEREST

The authors declare no conflict of interest.

### PEER REVIEW

The peer review history for this article is available at https://publons.com/publon/10.1002/brb3.2579

## Supporting information

SUPPORTING INFORMATION

SUPPORTING INFORMATION

## Data Availability

The data that support the findings of this study are available from the corresponding author upon reasonable request.
