# Supplementary figures and images for: Quality of life predicts rehabilitation prognosis in Parkinson's disease patients: Factors influence rehabilitation prognosis
Source: Brain Behav. 2022 Apr 16;12(5):e2579. doi: 10.1002/brb3.2579 (PMC9120870; doi:10.1002/brb3.2579)

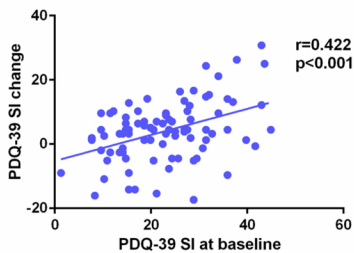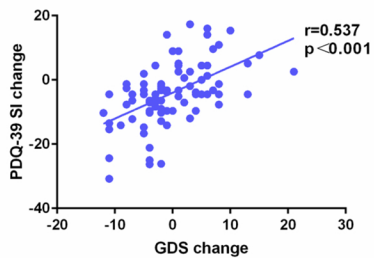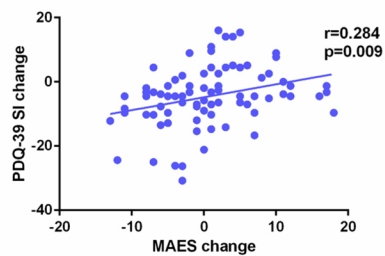

Supplement: Supplementary file 1 — SUPPORTING INFORMATION [file BRB3-12-e2579-s002.pdf]
