# Supplementary material for: Quality of life predicts rehabilitation prognosis in Parkinson's disease patients: Factors influence rehabilitation prognosis
Source: Brain Behav. 2022 Apr 16;12(5):e2579. doi: 10.1002/brb3.2579 (PMC9120870; doi:10.1002/brb3.2579)
Supplement: Supplementary file 2 — SUPPORTING INFORMATION [file BRB3-12-e2579-s001.docx]

Appendix.1. Correlation between PDQ-39 SI changes and PDQ-39 SI at baseline, GDS Score changes, MAES Score changes

PDQ-39 SI changes were significantly associated with the PDQ-39 SI at baseline and were significantly associated with GDS score changes and MAES score changes after the 3-month follow-up.
